# From acute diagnosis to longitudinal risk stratification: a paradigm shift in the clinical role of cardiac biomarkers

**DOI:** 10.3389/fcvm.2026.1778456

**Published:** 2026-03-13

**Authors:** Hong Zheng, Xue Li, Li-Jiao Guo, Guang-Ling Ji, Hong-Tao Liu, Yue Zheng, Jie Zhou

**Affiliations:** 1 Department of Clinical Laboratory, The Affiliated Hospital of Changchun University of Chinese Medicine, Changchun, China; 2 Department of Gastroenterology and Digestive Endoscopy Center, The Second Hospital of Jilin University, Changchun, China; 3 Administrative Office, The First Clinical Hospital of Jilin Provincial Academy of Chinese Medicine Sciences, Changchun, China; 4 Department of Geriatrics, The First Hospital of Jilin University, Changchun, China; 5 Cardiology Center, The Affiliated Hospital of Changchun University of Chinese Medicine, Changchun, China

**Keywords:** cardiac biomarkers, cardiovascular disease, multi-omics, precision medicine, preventive cardiology, risk stratification

## Abstract

This perspective examines the evolving role of cardiac biomarkers from acute diagnostic tools to integral components of longitudinal risk stratification and cardiovascular disease management. Evidence from cohort studies, clinical trials, and high-sensitivity assays demonstrates that biomarkers reflecting myocardial injury, hemodynamic stress, inflammation, fibrosis, and metabolic dysfunction can inform prevention, early detection, acute care, and chronic monitoring. Key implementation strategies include multi-biomarker panels, serial measurements, multi-omics integration, and artificial intelligence-based risk modeling. Challenges such as assay standardization, clinical interpretation, and cost-effectiveness are critically evaluated. Overall, this framework highlights the potential for biomarker-guided approaches to promote more preventive, precise, and patient-centered cardiovascular care.

## 1 Introduction

Cardiovascular disease (CVD) continues to be a primary cause of death and disability worldwide, presenting a persistent and substantial public health challenge (1). This burden underscores the need for management strategies that address CVD across its entire disease spectrum rather than focusing solely on acute events (2). Established cardiac biomarkers, particularly cardiac troponin (cTn) and B-type natriuretic peptide (BNP/NT-proBNP), have long served essential roles in clinical practice (3). Traditionally, their clinical use has predominantly focused on diagnosing acute cardiovascular conditions—such as myocardial infarction or acute heart failure—and on offering limited short-term prognostic evaluation (4). This reactive, event-centered approach provides limited insight into early disease processes and offers insufficient support for long-term risk stratification or primary prevention (5).

Concurrently, medical practice is undergoing a broader transformation from reactive disease treatment toward a proactive model grounded in Predictive, Preventive, Personalized, and Participatory (4P) medicine (6). This shift requires clinical tools capable of identifying risk at earlier stages, monitoring disease trajectories over time, and guiding intervention across different phases of disease progression (4, 6). Within this evolving framework, the clinical function of cardiac biomarkers is a significant conceptual transition.

**Figure 1:**
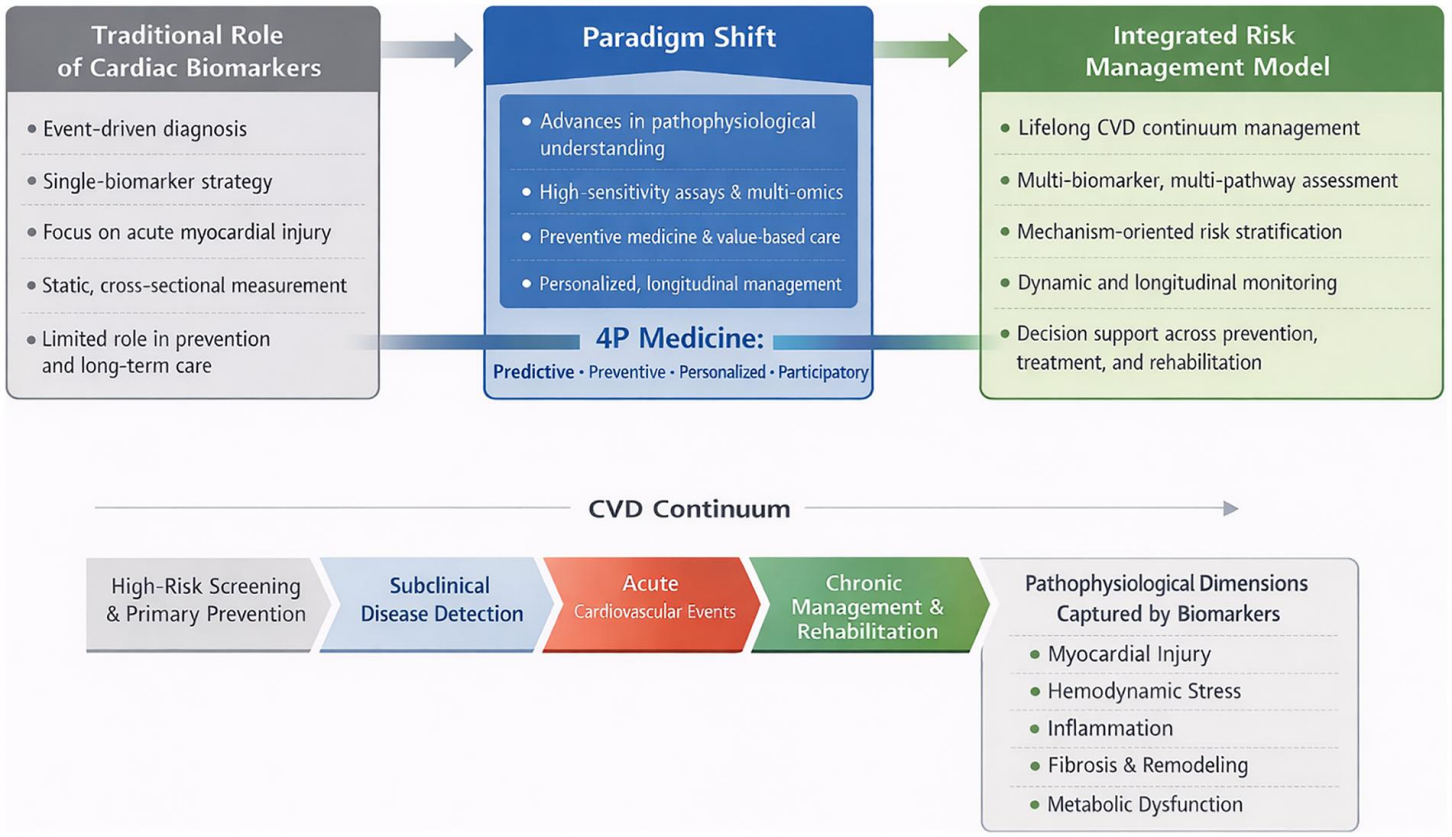
Paradigm shift in the role of cardiac biomarkers across the cardiovascular disease continuum. This figure illustrates the transition from an event-centered use of cardiac biomarkers toward a longitudinal, risk-based management framework. Biomarker applications are mapped across four major stages: high-risk screening and primary prevention, subclinical disease detection, acute diagnosis and risk stratification, and chronic disease management and rehabilitation. At each stage, biomarkers reflect distinct underlying pathophysiological processes, including myocardial injury, hemodynamic stress, inflammation, fibrosis, and metabolic dysfunction, thereby supporting stage-specific clinical decision-making.

This transition represents a paradigm shift. Rather than serving solely as diagnostic indicators of acute events, cardiac biomarkers are increasingly deployed as dynamic, multidimensional tools for integrated risk management throughout the full CVD continuum (Figure 1). This continuum encompasses the high-risk phase (screening and primary prevention), the subclinical phase (detection of early organ injury), the acute phase (rapid diagnosis and initial risk stratification), and the chronic or rehabilitative phase (treatment response monitoring, prognosis, and secondary prevention) (7). The growing availability of biomarkers that reflect myocardial stress, fibrosis, inflammation, and metabolic dysfunction has further expanded the scope of cardiovascular risk evaluation (7, 8).

Ultimately, contemporary cardiac biomarkers are increasingly positioned as core components of modern CVD management (4). By capturing distinct pathophysiological processes at different disease stages, these biomarkers help bridge underlying biological mechanisms with objective risk assessment and individualized clinical decision-making (4, 9). This integration supports a transition toward a more anticipatory, precise, and systematically organized model of cardiovascular care.

This perspective defines the proposed “paradigm shift” as a coordinated transition in three interrelated dimensions. It is crucial to clarify that this article adopts a narrative perspective rather than constituting a systematic review. The referenced literature was selected to exemplify pivotal conceptual advances, landmark clinical trials, and pertinent population-based studies within biomarker-guided risk management and does not constitute a comprehensive summary of the entire evidence base. The first dimension involves a fundamental change in the application of biomarkers, from tools for discrete event detection to instruments for longitudinal, dynamic risk surveillance. The second dimension concerns a shift in analytical strategy, from assessing individual biomarkers in isolation toward integrating multi-marker profiles that reflect complementary pathophysiological mechanisms. The third dimension represents an evolution in clinical intent, from a reactive model focused on diagnosis toward a proactive model prioritizing risk prediction, preventive intervention, and personalized treatment optimization. Collectively, these dimensions form a unified conceptual framework that distinguishes modern, biomarker-guided cardiovascular care from traditional diagnostic approaches.

To ensure the precision of this framework, it is essential to acknowledge a critical epistemological caveat that underpins the entire discussion. The proactive model proposed here is fundamentally built upon the capacity of biomarkers to stratify risk. Therefore, before elaborating on the applications of this paradigm, a clear operational distinction should be made. Clinical validity refers to a biomarker's ability to provide independent or incremental prognostic information beyond established risk factors. In contrast, clinical utility requires evidence that acting on that information leads to improved patient-centered outcomes. In many domains discussed in this perspective, the current evidence base more robustly supports clinical validity than definitive outcome improvement through biomarker-guided intervention. Consequently, any subsequent references to prevention or treatment optimization should be interpreted primarily within the context of risk stratification potential-identifying individuals or populations at higher likelihood of future events-rather than implying proven strategies for modifying those outcomes. This distinction safeguards against overinterpretation and aligns the discourse with the current standards of evidence-based medicine (10).

## 2 Theoretical basis and driving forces of the paradigm shift

The evolution in the role of cardiac biomarkers in CVD management reflects a broader transformation in modern medicine. This paradigm shift arises from multiple, interrelated developments and marks a transition from reactive, event-driven care toward proactive, mechanism-based, and integrated disease management.

### 2.1 Advances in pathophysiological insight

Traditionally, cardiac biomarkers such as cardiac troponin were primarily used to detect acute myocardial injury, particularly myocardial necrosis. Contemporary research, however, recognizes CVD as a continuous process characterized by interacting pathophysiological mechanisms, including myocardial stress, fibrosis, chronic low-grade inflammation, and metabolic dysregulation (11). This expanded understanding has driven the development and clinical adoption of a broader biomarker spectrum. Natriuretic peptides reflect myocardial wall stress and volume overload. Soluble suppression of tumorigenicity 2 (sST2) and procollagen type III N-terminal peptide provide insight into myocardial fibrosis and remodeling (12–14). High-sensitivity C-reactive protein (hs-CRP) extends beyond a general marker of systemic inflammation; it specifically reflects chronic low-grade vascular inflammation, which directly contributes to endothelial dysfunction and atherogenesis (15–17). Elevated hs-CRP levels are consistently associated with a higher risk of major adverse cardiovascular events, including myocardial infarction, stroke, and cardiovascular mortality, independent of traditional risk factors (18). Crucially, hs-CRN provides incremental prognostic value beyond markers of myocardial injury and hemodynamic stress, thereby capturing the distinct contribution of inflammatory pathways to cardiovascular risk—a dimension not assessed by cardiac-specific biomarkers alone.

Overall, these markers enable clinicians to monitor disease progression across its stages, moving beyond acute diagnosis toward continuous risk assessment. These advances collectively support a transition from an event-centered interpretation of biomarkers toward the continuous monitoring of underlying disease mechanisms.

### 2.2 Technological progress in biomarker measurement

Technological advances in biomarker measurement have been central to this paradigm shift. High-sensitivity assays can now detect biomarkers at very low concentrations, uncovering subclinical myocardial injury that was previously undetectable (19). In parallel, multi-omics technologies—particularly proteomics and metabolomics— enable systematic discovery of novel biomarker signatures and provide deeper insight into disease-specific molecular networks (20). These approaches enhance mechanistic understanding and support more refined risk stratification. Point-of-care testing platforms further expand clinical applicability by enabling rapid biomarker assessment in diverse settings, including primary care, emergency departments, and resource-limited environments (21). Collectively, these technological developments allow biomarkers to be applied earlier, measured more frequently, and used across a wider range of clinical contexts than was previously feasible.

### 2.3 Evolving demands in clinical practice

Clinical practice increasingly requires tools that support long-term cardiovascular risk management rather than solely acute diagnosis. Several needs drive this shift (22–25). First, precise risk stratification is required to identify high-risk individuals before clinical symptoms develop. Second, early detection of potentially reversible pathological changes enables timely intervention. Third, biomarker profiles can inform individualized treatment selection. Finally, longitudinal monitoring supports assessment of therapeutic response and adjustment of management strategies over time. These demands reflect a broader movement toward personalized and evidence-based care. Biomarkers are increasingly expected to guide decision-making across the entire disease trajectory, rather than serving a single diagnostic purpose.

### 2.4 Influence of preventive medicine and value-based care

Broader healthcare trends further reinforce this transformation. Preventive medicine emphasizes early risk detection and primary prevention, encouraging biomarker use before overt disease develops (4). At the same time, value-based care prioritizes improved long-term outcomes while optimizing healthcare resources (26). Biomarkers contribute to these goals by improving risk prediction accuracy, supporting targeted interventions, and reducing unnecessary testing or treatment. In this context, biomarker-guided strategies align clinical decision-making with both patient-centered outcomes and healthcare system sustainability (4, 10). Together, these advances in pathophysiology, technology, clinical practice, and healthcare priorities explain why cardiac biomarkers are increasingly used for ongoing cardiovascular risk management, rather than only for event-based diagnosis.

In summary, this paradigm shift results from converging advances in disease biology, measurement technology, clinical expectations, and healthcare priorities. Cardiac biomarkers are no longer limited to diagnostic confirmation of acute events. Instead, they have become essential tools for predicting risk, guiding prevention, and managing CVD across its entire course.

## 3 Core application scenarios and biomarker profiles in the new paradigm

This integrated approach is necessitated by the pathophysiological complexity and temporal evolution of cardiovascular disease. Consequently, biomarkers should be evaluated based on their distinct biological mechanisms and stage-specific clinical validities. This rationale leads to their classification into dedicated categories, such as those indicative of myocardial injury [e.g., high-sensitivity cardiac troponin (hs-cTn)], hemodynamic stress (e.g., BNP/NT-proBNP), inflammation (e.g., hs-CRP), fibrosis and remodeling (e.g., sST2, galectin-3), and cellular stress (e.g., growth differentiation factor 15). Accordingly, specific biomarker profiles align with particular disease stages and underlying pathophysiological mechanisms (Supplementary Table S1). Moving beyond isolated or supplementary use, biomarkers are increasingly combined and measured longitudinally. This approach enables continuous risk evaluation and supports dynamic clinical decision-making. This systematic application of biomarkers, as demonstrated across the following scenarios, provides a structured and clinically applicable framework that aligns biomarker use with disease stage, encompassing the full spectrum from prevention and diagnosis to treatment and long-term follow-up.

### 3.1 Primary prevention and high-risk population screening

In primary prevention, biomarkers help identify individuals with subclinical myocardial injury or persistent cardiac stress before symptoms manifest. Elevated hs-cTn reflects minor cardiomyocyte injury or turnover, while BNP/NT-proBNP indicates myocardial wall stress and volume overload (3, 27). Hs-CRP provides information on low-grade systemic inflammation (28). Even modest and sustained elevations in these biomarkers are independently associated with increased cardiovascular risk in asymptomatic individuals. In preventive cardiology, biomarker-based risk stratification is particularly valuable for identifying high-risk individuals who may not be recognized by conventional risk assessment models. The integration of hs-cTn, natriuretic peptides, and inflammatory markers facilitates the earlier detection of not only myocardial injury and subclinical cardiac stress but also underlying vascular inflammation (29–31). This comprehensive biomarker profile enables a more precise estimation of cardiovascular risk than traditional factors alone (3). Consequently, this approach supports early lifestyle modification and preventive pharmacological interventions in populations that may otherwise be considered low risk based on traditional factors.

However, despite these promising associations, the implementation of biomarker-based screening in primary prevention is not without potential harms. High-sensitivity assays may detect low-level biomarker elevations unrelated to modifiable cardiovascular pathology, particularly in populations such as older adults or those with chronic kidney disease, where baseline troponin and natriuretic peptide levels are often chronically elevated (32, 33). Such findings may lead to false-positive classifications, overestimation of risk, and downstream consequences including unnecessary diagnostic testing, increased healthcare utilization, potential overtreatment, and patient anxiety (34). Moreover, indiscriminate screening risks exacerbating health disparities, especially when access to confirmatory testing and follow-up care is unevenly distributed. Therefore, for biomarker-informed screening to be both clinically effective and equitable, implementation should occur within structured risk assessment frameworks and supported by rigorous cost-effectiveness, calibration and equity analyses (4, 34).

### 3.2 Early disease detection and subclinical management

This stage focuses on identifying structural and functional cardiac abnormalities before overt clinical disease develops. Biomarkers such as sST2 and galectin-3 reflect fibrotic and inflammatory processes involved in myocardial remodeling. Growth differentiation factor-15, a stress-responsive cytokine, is associated with early atherosclerosis and heart failure risk (35, 36). By revealing early pathological changes, these biomarkers facilitate timely intervention. They support proactive management of patients with occult coronary disease or early myocardial dysfunction, potentially slowing disease progression.

### 3.3 Rapid diagnosis and differential diagnosis of acute cardiovascular events

In acute care settings, biomarkers play a critical role in rapid diagnosis and triage. Serial measurements of hs-cTn using accelerated diagnostic algorithms are central to the early diagnosis of acute myocardial infarction (37–39). Emerging biomarkers, such as cardiac myosin-binding protein C, demonstrate faster release kinetics than traditional troponins. These characteristics may improve very early diagnosis and enhance decision-making in emergency departments, reducing delays to definitive treatment (40).

### 3.4 In-Hospital and post-discharge risk stratification and prognosis

Beyond diagnosis, biomarkers provide important prognostic information during hospitalization and after discharge. Elevated levels of hs-cTn, natriuretic peptides, and inflammatory markers are associated with increased risk of mortality and rehospitalization (41). Multi-marker strategies combine complementary biological information. For example, integrating markers of myocardial injury, hemodynamic stress, and inflammation improves risk stratification and helps identify patients who may benefit from intensified treatment or closer follow-up (42).

From a prognostic perspective, hs-CRP improves risk stratification in multi-marker panels by capturing systemic inflammation, a process closely associated with adverse cardiac remodeling and recurrent cardiovascular events (43). While hs-cTn indicates myocardial injury and natriuretic peptides reflect hemodynamic stress, hs-CRP provides complementary information on this distinct inflammatory pathway (44). Patients with persistently elevated hs-CRP levels after an acute cardiovascular event demonstrate significantly higher rates of rehospitalization and mortality, underscoring its specific utility for post-discharge risk assessment (45).

### 3.5 Chronic disease management: treatment guidance and monitoring

In chronic cardiovascular conditions, particularly heart failure, biomarkers are valuable for guiding therapy and monitoring response. Changes in natriuretic peptide levels reflect hemodynamic status and treatment effectiveness. A sustained reduction after initiation of guideline-directed medical therapy is associated with improved clinical outcomes (46, 47). Serial biomarker measurements support individualized treatment titration and enable a treat-to-target approach. Integrating biomarker data with clinical evaluation enables more nuanced therapeutic decision-making. Although reductions in natriuretic peptides correlate with favorable prognosis and some trials suggest benefit from biomarker-guided strategies, evidence for consistent improvement in hard clinical endpoints across heterogeneous populations remains inconclusive (48). Accordingly, serial biomarker monitoring is best regarded as a tool for enhanced risk stratification rather than a validated treat-to-target mandate (48).

### 3.6 Emerging applications: cardiotoxicity monitoring and cardiac rehabilitation

Biomarkers also play an expanding role in specialized clinical settings. In cardio-oncology, hs-cTn and natriuretic peptides are increasingly used to detect early chemotherapy-related cardiotoxicity, allowing timely implementation of cardioprotective strategies (49, 50). In cardiac rehabilitation, longitudinal biomarker trends may objectively reflect physiological improvement, such as reduced myocardial stress or favorable remodeling. These measurements can help tailor rehabilitation intensity and monitor treatment effectiveness (51, 52).

**Figure 2:**
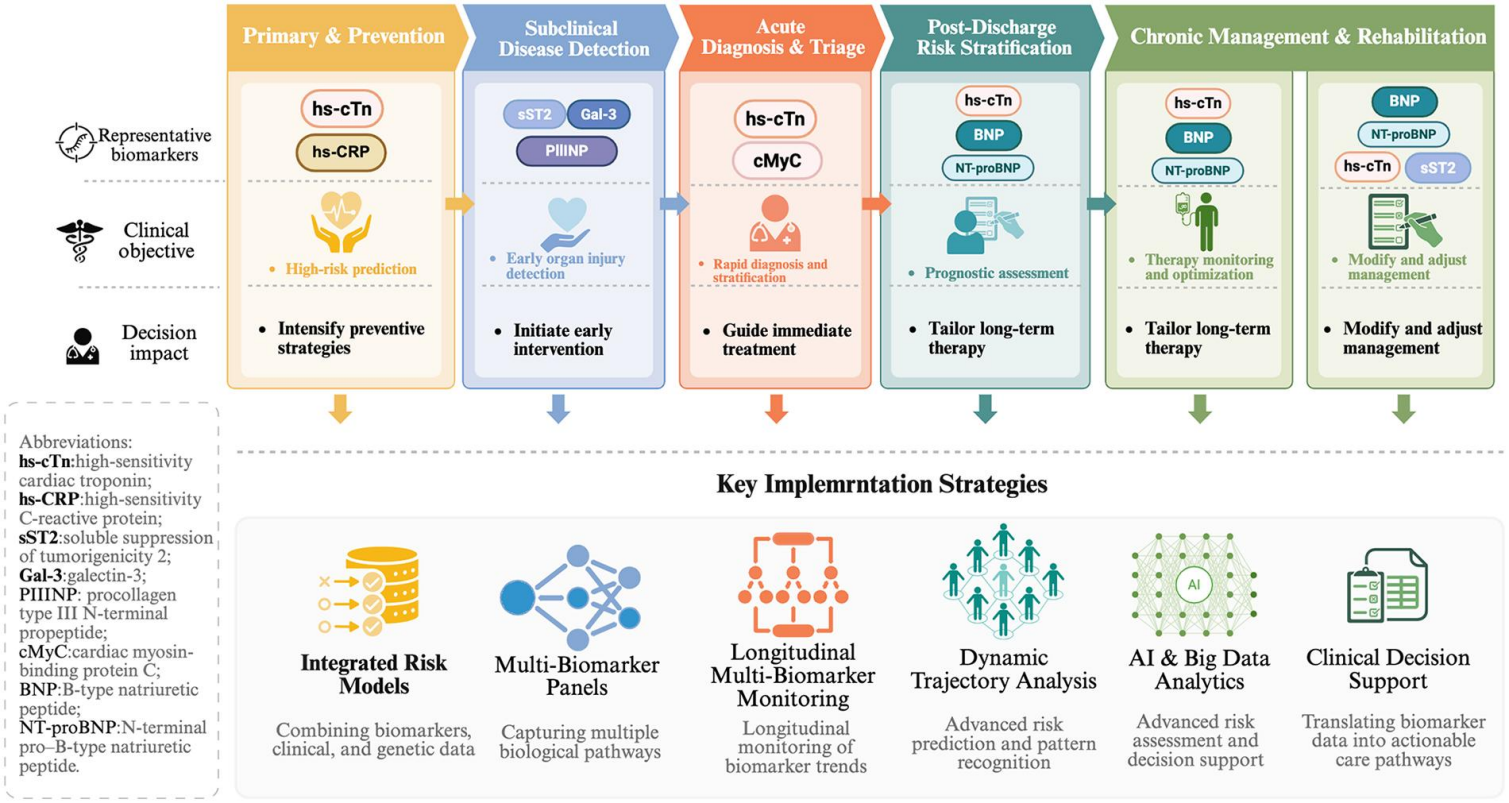
Stage-Specific applications and implementation strategies of cardiac biomarkers. This figure presents an integrated clinical pathway illustrating how multi-biomarker panels, serial measurements, and data-driven analytics can be incorporated into routine cardiovascular care. Biomarkers are used to support risk stratification, guide treatment selection and titration, monitor therapeutic response, and inform prognosis. The framework emphasizes longitudinal assessment and dynamic risk re-evaluation, providing a practical reference for implementing biomarker-based strategies in prevention, acute care, and chronic disease management.

In summary, biomarkers in the new paradigm are applied in a structured and stage-specific manner across the cardiovascular disease spectrum (Figure 2). By aligning biomarker categories with disease stage and clinical purpose, this framework provides clinicians with a practical reference for biomarker selection and interpretation. Integrated use of biomarkers across prevention, diagnosis, treatment, and follow-up supports a more proactive, individualized, and systematic approach to cardiovascular care.

## 4 Key strategies and tools for achieving the paradigm shift

To achieve this shift in cardiac biomarker use—from simple diagnostic aids to core parts of risk management—a systematic set of strategies and innovative tools is essential. This will make biomarkers more useful in practice and support cardiovascular care that is precise, adaptable, and well-coordinated.

### 4.1 Integrated risk assessment models

The clinical value of a single biomarker is inherently limited. Its utility increases substantially when combined with complementary sources of information. Integrating biomarkers with traditional risk factors (e.g., blood pressure, lipid profiles), as well as imaging findings and genetic data, enables the construction of multidimensional risk assessment models (53). These integrated models improve risk stratification by identifying individuals who may be underestimated by conventional scores alone. They also support the definition of individualized treatment thresholds, which is essential for precision prevention (54). For example, combining high-sensitivity cardiac troponin with polygenic risk scores has been shown to enhance the prediction of future cardiovascular events in asymptomatic populations (53, 55). Such integrated models are particularly valuable in preventive care. They support personalized risk assessment and enable earlier, targeted interventions based on individual mechanisms, moving beyond uniform, population-wide prevention approaches. Overall, integrated risk models provide a more comprehensive and clinically actionable representation of cardiovascular risk.

### 4.2 Dynamic monitoring and trajectory analysis

Single biomarker measurements offer only a snapshot of disease status. In contrast, changes in biomarker levels over time often provide more clinically meaningful information. Longitudinal monitoring allows clinicians to assess disease progression and treatment response more accurately (56). In heart failure, rising natriuretic peptide levels may signal impending clinical deterioration, whereas sustained reductions are often associated with therapeutic benefit and improved prognosis (57). By focusing on trajectories rather than isolated values, clinicians can detect risk earlier and adjust therapy in a timely manner (58). Thus, dynamic monitoring shifts biomarker use from static interpretation toward longitudinal risk assessment. While trajectory-based evaluation may enhance prognostic accuracy, further prospective studies are needed to confirm that routine, trajectory-guided adjustments consistently lead to improved clinical outcomes.

### 4.3 Multi-biomarker panel strategies

Cardiovascular disease is driven by multiple overlapping pathophysiological processes. No single biomarker can adequately capture this complexity. Multi-biomarker panels address this limitation by combining markers that reflect distinct biological pathways, including myocardial injury, hemodynamic stress, inflammation, and fibrosis (59). This approach improves diagnostic accuracy and supports disease phenotyping, such as distinguishing inflammatory-dominant from fibrotic-dominant disease patterns. It also facilitates more targeted therapeutic decisions, thereby advancing precision cardiovascular medicine (60, 61). Multi-biomarker strategies therefore offer a more complete and clinically informative assessment than single-marker approaches.

### 4.4 Artificial intelligence and big data analytics

The increasing volume of clinical, laboratory, and multi-omics data exceeds the capacity of traditional analytical methods. Artificial intelligence and machine learning provide tools to manage this complexity and extract clinically relevant patterns (62). Artificial intelligence can process and analyze large and complex datasets beyond the reach of traditional statistical tools (63). In cardiovascular research, these datasets often encompass electronic health records, repeated biomarker measurements, medical images, and multi-omics data from extensive population studies (64). It is important to note, however, that not all studies employing these advanced analytical methods utilize true longitudinal trajectory modeling (65, 66). While some analyses are based on cross-sectional biomarker features or single baseline measurements, others explicitly integrate repeated measures over time (65, 67). This distinction is critical, as trajectory-based models, which leverage machine learning algorithms to model non-linear associations and track changes in biomarkers longitudinally (68), are better positioned to capture dynamic risk. Yet, they also require more complex data infrastructure and rigorous validation to ensure clinical utility.

This capability supports dynamic, individualized risk prediction that updates with new data, moving beyond static, one-time assessments (69). Consequently, these methods can identify novel biomarker signatures, optimize risk prediction models, and refine clinical decision pathways using large-scale real-world data (70). Importantly, studies have shown that machine learning models integrating biomarker data—particularly when leveraging repeated or longitudinal measurements—may improve discrimination metrics compared with conventional risk scores in selected datasets; however, such improvements are context dependent and have not been consistently replicated across diverse populations or clinical settings (62, 70, 71).

Recent population-based studies demonstrate that artificial intelligence-assisted biomarker models can be implemented at the health-system level to monitor cardiovascular risk in real time across large patient populations (72, 73). These models employ continuous learning frameworks, through which incoming clinical and laboratory data iteratively refine risk predictions (74). This process facilitates timely preventive interventions and supports personalized disease management.

The scalability of this approach is being confirmed. Recent large population-based studies from 2025 and early 2026 have not only demonstrated methodological feasibility and scalability within retrospective or observational datasets, but they also further illustrated the practicality of deploying artificial intelligence-assisted biomarker integration at the health system level for population-scale monitoring (75–78). Despite these advances, widespread real-world deployment with demonstrated clinical impact remains an ongoing area of investigation rather than an established standard of care. Collectively, these findings underscore the increasing practicality of incorporating machine learning-based biomarker models into routine clinical practice for real-time cardiovascular risk monitoring.

Before clinical implementation, machine learning-based biomarker models should meet several essential standards to ensure scientific robustness and ethical integrity. First, they should undergo rigorous external validation in independent and demographically diverse populations to confirm generalizability (79, 80). Second, models should demonstrate adequate calibration and transparently report discrimination metrics, including confidence intervals and decision-curve analyses where appropriate (79, 80). Third, algorithmic fairness should be systematically assessed to detect potential bias across sex, age, ethnicity, socioeconomic status, and comorbidity subgroups (80, 81). Fourth, continuous post-deployment surveillance is required to identify model drift and performance degradation over time (82). Fifth, and most critically, prospective evaluation of their impact on clinical decision-making and patient outcomes is necessary to establish true clinical utility (83). Without adherence to these safeguards, predictive performance alone cannot justify routine clinical integration.

In summary, artificial intelligence-driven biomarker analytics represent a powerful but methodologically demanding extension of precision cardiovascular medicine. Their promise lies in enhancing risk stratification and supporting dynamic decision-making; however, their clinical legitimacy ultimately depends on rigorous validation, transparent reporting, equitable performance, and demonstrable outcome benefit. Thoughtful integration of these safeguards is essential to translate computational innovation into meaningful improvements in cardiovascular care.

## 5 Challenges and future directions

Despite substantial progress, several barriers continue to limit the routine clinical implementation of biomarker-guided cardiovascular care. While biomarkers have advanced cardiovascular care, their routine use in clinical practice remains limited by structural, analytical, and implementation challenges. Addressing these challenges is essential for translating conceptual advances into sustained clinical benefit.

### 5.1 Key challenges

Four major challenges currently limit broader implementation. One major challenge is pre-analytical and analytical variability. Differences in sample collection, processing, and assay performance can significantly affect biomarker measurements. Without standardized protocols and validated reference ranges, results may not be comparable across clinical settings (84, 85). A second challenge lies in clinical interpretation. Biomarker levels can be influenced by comorbid conditions such as chronic kidney disease, obesity, or atrial fibrillation. These factors may reduce specificity for cardiac pathology and complicate clinical decision-making (86). Third, robust health economic evidence is often lacking. Cost-effectiveness analyses are needed to justify the broader use of biomarker-based screening and longitudinal monitoring compared with conventional strategies (87). Finally, barriers to clinical translation persist. These include delayed updates to clinical guidelines, variable clinician familiarity with newer biomarkers, and limited integration of biomarker data into electronic health records and clinical workflows (88).

### 5.2 Future directions

Future progress will require coordinated efforts in several key areas. First, multi-omics technologies such as proteomics and metabolomics are expected to yield more specific biomarkers aligned with distinct disease mechanisms and stages (89). Second, individualized biomarker thresholds should be developed. Adjusting cut-off values for factors such as age, sex, renal function, and comorbidities may improve both diagnostic and prognostic accuracy (90). Third, digital integration should be strengthened. Embedding serial biomarker data into electronic health records and clinical decision support systems would enable dynamic risk assessment and personalized treatment guidance (91, 92). Finally, prospective biomarker-guided interventional trials are needed. Such studies are essential to determine whether biomarker-driven strategies can improve hard clinical outcomes, including mortality and hospitalization rates (93).

Rather than proposing novel biomarkers, this perspective's key contribution lies in rethinking how both established and emerging biomarkers can be deployed along longitudinal, stage-specific care pathways. This integrated framework moves beyond describing biomarker-disease associations toward actionable, system-level strategies. These strategies align biomarker application with preventive cardiology, treatment individualization, and sustainable health system design. The novelty of this paradigm lies in its integrated approach, not in discovering single biomarkers. It systematically embeds biomarkers into longitudinal care pathways, connecting prevention, treatment optimization, and value-based healthcare delivery.

Addressing these challenges and pursuing these strategies will help turn biomarkers from research tools into practical parts of cardiovascular care. This will support more precise, evidence-based, and personalized patient management.

## 6 Summary

The clinical role of cardiac biomarkers has evolved substantially. Once used mainly for the diagnosis of acute events, they are now integral to comprehensive and personalized cardiovascular risk assessment across all stages of disease. This evolution reflects a broader shift from reactive diagnosis to proactive risk prediction, from isolated measurements to longitudinal monitoring, and from uniform treatment strategies to individualized management.

Realizing the full potential of this paradigm will require close collaboration among laboratory medicine, cardiology, epidemiology, and data science. Key priorities include assay standardization, effective integration of biomarker data into clinical workflows, and the generation of high-quality evidence to guide practice. Through these efforts, biomarker-guided strategies can support a more preventive, precise, and patient-centered model of cardiovascular care, with the ultimate goal of improving long-term outcomes.

## Data Availability

The original contributions presented in the study are included in the article/Supplementary Material, further inquiries can be directed to the corresponding author.

## References

[1] Yang H Ma Q Han L Liu H. A global prediction of cardiovascular disease from 2020 to 2030. Front Cardiovasc Med. (2025) 12:1462705. 10.3389/fcvm.2025.1462705 40860359 PMC12375611

[2] Victor G Shishani K Vellone E Froelicher ES. The global burden of cardiovascular disease in adults: a mapping review. J Cardiovasc Nurs. (2025). 10.1097/JCN.0000000000001200 40179360

[3] Everett BM Zeller T Glynn RJ Ridker PM Blankenberg S. High-sensitivity cardiac troponin I and B-type natriuretic peptide as predictors of vascular events in primary prevention: impact of statin therapy. Circulation. (2015) 131(21):1851–60. 10.1161/CIRCULATIONAHA.114.014522 25825410 PMC4444427

[4] Neumann JT de Lemos JA Apple FS Leong DP. Cardiovascular biomarkers for risk stratification in primary prevention. Eur Heart J. (2025) 46(39):3823–43. 10.1093/eurheartj/ehaf517 40795138

[5] Neumann JT Twerenbold R Weimann J Ballantyne CM Benjamin EJ Costanzo S Prognostic value of cardiovascular biomarkers in the population. JAMA. (2024) 331(22):1898–909. 10.1001/jama.2024.5596 38739396 PMC11091824

[6] Sagner M McNeil A Puska P Auffray C Price ND Hood L The P4 health Spectrum - A predictive, preventive, personalized and participatory Continuum for promoting healthspan. Prog Cardiovasc Dis. (2017) 59(5):506–21. 10.1016/j.pcad.2016.08.002 27546358

[7] Thupakula S Nimmala SSR Ravula H Chekuri S Padiya R. Emerging biomarkers for the detection of cardiovascular diseases. Egypt Heart J. (2022) 74(1):77. 10.1186/s43044-022-00317-2 36264449 PMC9584006

[8] Kurt B Rex K Reugels M Fordyce CB Fudim M Sharma A Inflammatory biomarkers in heart failure: clinical perspectives on hsCRP, IL-6 and emerging candidates. Curr Heart Fail Rep. (2025) 22(1):35. 10.1007/s11897-025-00710-3 41196450 PMC12592283

[9] Heidenreich PA Bozkurt B Aguilar D Allen LA Byun JJ Colvin MM 2022 AHA/ACC/HFSA guideline for the management of heart failure: a report of the American College of Cardiology/American Heart Association joint committee on clinical practice guidelines. Circulation. (2022) 145(18):e895–e1032. 10.1161/CIR.0000000000001063 35363499

[10] Morrow DA de Lemos JA. Benchmarks for the assessment of novel cardiovascular biomarkers. Circulation. (2007) 115(8):949–52. 10.1161/CIRCULATIONAHA.106.683110 17325253

[11] Sharif S Van der Graaf Y Cramer MJ Kapelle LJ de Borst GJ Visseren FLJ Low-grade inflammation as a risk factor for cardiovascular events and all-cause mortality in patients with type 2 diabetes. Cardiovasc Diabetol. (2021) 20(1):220. 10.1186/s12933-021-01409-0 34753497 PMC8579639

[12] Ulziisaikhan G Khurelbaatar MU Khorloo C Khasag A Unurjargal T. Utilizing natriuretic peptides for predicting heart failure risk following myocardial infarction. Cardiovasc Endocrinol Metab. (2025) 14(3):e00338. 10.1097/XCE.0000000000000338 40672529 PMC12266924

[13] McCarthy CP Januzzi JL Jr. Soluble ST2 in heart failure. Heart Fail Clin. (2018) 14(1):41–8. 10.1016/j.hfc.2017.08.005 29153199

[14] Mansour IN Bress AP Groo V Ismail S Wu G Patel SR Circulating procollagen type III N-terminal peptide and mortality risk in African Americans with heart failure. J Card Fail. (2016) 22(9):692–9. 10.1016/j.cardfail.2015.12.016 26721774 PMC4917490

[15] Libby P Ridker PM. Inflammation and atherosclerosis: role of C-reactive protein in risk assessment. Am J Med. (2004) 116(Suppl 6A):9S–16. 10.1016/j.amjmed.2004.02.006 15050187

[16] Devaraj S Yun JM Adamson G Galvez J Jialal I. C-reactive protein impairs the endothelial glycocalyx resulting in endothelial dysfunction. Cardiovasc Res. (2009) 84(3):479–84. 10.1093/cvr/cvp249 19620133 PMC2777951

[17] Badimon L Peña E Arderiu G Padró T Slevin M Vilahur G C-Reactive protein in atherothrombosis and angiogenesis. Front Immunol. (2018) 9:430. 10.3389/fimmu.2018.00430 29552019 PMC5840191

[18] Fonseca FA Izar MC. High-Sensitivity C-reactive protein and cardiovascular disease across countries and ethnicities. Clinics (Sao Paulo). (2016) 71(4):235–42. 10.6061/clinics/2016(04)11 27166776 PMC4825196

[19] Xu S Liu H Bai Y. Highly sensitive and multiplexed mass spectrometric immunoassay techniques and clinical applications. Anal Bioanal Chem. (2022) 414(18):5121–38. 10.1007/s00216-022-03945-4 35165779

[20] Birhanu AG. Mass spectrometry-based proteomics as an emerging tool in clinical laboratories. Clin Proteomics. (2023) 20(1):32. 10.1186/s12014-023-09424-x 37633929 PMC10464495

[21] Manoharan Nair Sudha Kumari S Thankappan Suryabai X. Sensing the future-frontiers in biosensors: exploring classifications, principles, and recent advances. ACS Omega. (2024) 9(50):48918–87. 10.1021/acsomega.4c07991 39713646 PMC11656264

[22] Liu T Krentz A Lu L Curcin V. Machine learning based prediction models for cardiovascular disease risk using electronic health records data: systematic review and meta-analysis. Eur Heart J Digit Health. (2024) 6(1):7–22. 10.1093/ehjdh/ztae080 39846062 PMC11750195

[23] Gruson D Peranio C Štaraitė A Hobbs R Borch-Johnsen K Ademi Z Highlights from the manifesto on early detection and diagnosis of cardiovascular disease: the role of laboratory tests and emerging technologies. EJIFCC. (2025) 36(1):9–11. 40061066 PMC11886626

[24] Nazir A Nazir A Afzaal U Aman S Sadiq SUR Akah OZ Advancements in biomarkers for early detection and risk stratification of cardiovascular diseases-A literature review. Health Sci Rep. (2025) 8(5):e70878. 10.1002/hsr2.70878 40432692 PMC12106349

[25] Saba PS Al Kindi S Nasir K. Redefining cardiovascular risk assessment as a Spectrum: from binary to continuous. J Am Coll Cardiol. (2024) 83(5):574–6. 10.1016/j.jacc.2023.11.026 38296401

[26] Porter ME. What is value in health care? N Engl J Med. (2010) 363(26):2477–81. 10.1056/NEJMp1011024 21142528

[27] Kistorp C Raymond I Pedersen F Gustafsson F Faber J Hildebrandt P. N-terminal pro-brain natriuretic peptide, C-reactive protein, and urinary albumin levels as predictors of mortality and cardiovascular events in older adults. JAMA. (2005) 293(13):1609–16. 10.1001/jama.293.13.1609 15811980

[28] Yeh ET. High-sensitivity C-reactive protein as a risk assessment tool for cardiovascular disease. Clin Cardiol. (2005) 28(9):408–12. 10.1002/clc.4960280905 16250263 PMC6654463

[29] Willeit P Welsh P Evans JDW Tschiderer L Boachie C Jukema JW High-Sensitivity cardiac troponin concentration and risk of first-ever cardiovascular outcomes in 154,052 participants. J Am Coll Cardiol. (2017) 70(5):558–68. 10.1016/j.jacc.2017.05.062 28750699 PMC5527070

[30] Natriuretic Peptides Studies Collaboration, Willeit P Kaptoge S Welsh P Butterworth AS Chowdhury R Natriuretic peptides and integrated risk assessment for cardiovascular disease: an individual-participant-data meta-analysis. Lancet Diabetes Endocrinol. (2016) 4(10):840–9. 10.1016/S2213-8587(16)30196-6 27599814 PMC5035346

[31] Ridker PM Danielson E Fonseca FA Genest J Gotto AM Jr Kastelein JJ Rosuvastatin to prevent vascular events in men and women with elevated C-reactive protein. N Engl J Med. (2008) 359(21):2195–207. 10.1056/NEJMoa0807646 18997196

[32] Tagore R Ling LH Yang H Daw HY Chan YH Sethi SK. Natriuretic peptides in chronic kidney disease. Clin J Am Soc Nephrol. (2008) 3(6):1644–51. 10.2215/CJN.00850208 18632852 PMC2572269

[33] Sedighi SM Prud'Homme P Ghachem A Lepage S Nguyen M Fulop T Increased level of high-sensitivity cardiac troponin T in a geriatric population is determined by comorbidities compared to age. Int J Cardiol Heart Vasc. (2019) 22:187–91. 10.1016/j.ijcha.2019.02.015 30963093 PMC6437284

[34] Wiedermann CJ Piccoliori G Engl A Hager von Strobele-Prainsack D. Predictive biomarkers for asymptomatic adults: opportunities, risks, and guidance for general practice. Diagnostics (Basel). (2026) 16(2):196. 10.3390/diagnostics16020196 41594172 PMC12839772

[35] Gawor M Śpiewak M Janas J Kożuch K Wróbel A Mazurkiewicz Ł The usefulness of sST2 and galectin-3 as novel biomarkers for better risk stratification in hypertrophic cardiomyopathy. Kardiol Pol. (2017) 75(10):997–1004. 10.5603/KP.a2017.0118 28612913

[36] Tuegel C Katz R Alam M Bhat Z Bellovich K de Boer I GDF-15, galectin 3, soluble ST2, and risk of mortality and cardiovascular events in CKD. Am J Kidney Dis. (2018) 72(4):519–28. 10.1053/j.ajkd.2018.03.025 29866459 PMC6153047

[37] Collet JP Thiele H Barbato E Barthélémy O Bauersachs J Bhatt DL 2020 ESC guidelines for the management of acute coronary syndromes in patients presenting without persistent ST-segment elevation. Eur Heart J. (2021) 42(14):1289–367. 10.1093/eurheartj/ehaa575 32860058

[38] Reichlin T Hochholzer W Bassetti S Steuer S Stelzig C Hartwiger S Early diagnosis of myocardial infarction with sensitive cardiac troponin assays. N Engl J Med. (2009) 361(9):858–67. 10.1056/NEJMoa0900428 19710484

[39] Twerenbold R Neumann JT Sörensen NA Ojeda F Karakas M Boeddinghaus J Prospective validation of the 0/1-h algorithm for early diagnosis of myocardial infarction. J Am Coll Cardiol. (2018) 72(6):620–32. 10.1016/j.jacc.2018.05.040 30071991

[40] Kaier TE Twerenbold R Puelacher C Marjot J Imambaccus N Boeddinghaus J Direct comparison of cardiac myosin-binding protein C with cardiac troponins for the early diagnosis of acute myocardial infarction. Circulation. (2017) 136(16):1495–508. 10.1161/CIRCULATIONAHA.117.028084 28972002 PMC5642333

[41] Zhang ZY Wang XY Hou CC Liu HB Lyu L Chen ML Multiple biomarkers risk score for accurately predicting the long-term prognosis of patients with acute coronary syndrome. J Geriatr Cardiol. (2025) 22(7):656–67. 10.26599/1671-5411.2025.07.001 40896571 PMC12394956

[42] Ullah A Sajid S Qureshi M Kamran M Anwaar MA Naseem MA Novel biomarkers and the multiple-marker approach in early detection, prognosis, and risk stratification of cardiac diseases: a narrative review. Cureus. (2023) 15(7):e42081. 10.7759/cureus.42081 37602073 PMC10434821

[43] Sabatine MS Morrow DA de Lemos JA Gibson CM Murphy SA Rifai N Multimarker approach to risk stratification in non-ST elevation acute coronary syndromes: simultaneous assessment of troponin I, C-reactive protein, and B-type natriuretic peptide. Circulation. (2002) 105(15):1760–3. 10.1161/01.cir.0000015464.18023.0a 11956114

[44] Ray KK Cannon CP Cairns R Morrow DA Ridker PM Braunwald E. Prognostic utility of apoB/AI, total cholesterol/HDL, non-HDL cholesterol, or hs-CRP as predictors of clinical risk in patients receiving statin therapy after acute coronary syndromes: results from PROVE IT-TIMI 22. Arterioscler Thromb Vasc Biol. (2009) 29(3):424–30. 10.1161/ATVBAHA.108.181735 19122170

[45] Mani P Puri R Schwartz GG Nissen SE Shao M Kastelein JJP Association of initial and serial C-reactive protein levels with adverse cardiovascular events and death after acute coronary syndrome: a secondary analysis of the VISTA-16 trial. JAMA Cardiol. (2019) 4(4):314–20. 10.1001/jamacardio.2019.0179 30840024 PMC6484785

[46] Januzzi JL Jr Ahmad T Mulder H Coles A Anstrom KJ Adams KF Natriuretic peptide response and outcomes in chronic heart failure with reduced ejection fraction. J Am Coll Cardiol. (2019) 74(9):1205–17. 10.1016/j.jacc.2019.06.055 31466618 PMC6719723

[47] Persson H Erntell H Eriksson B Johansson G Swedberg K Dahlström U. Improved pharmacological therapy of chronic heart failure in primary care: a randomized study of NT-proBNP guided management of heart failure–SIGNAL-HF (Swedish intervention study–guidelines and NT-proBNP AnaLysis in heart failure). Eur J Heart Fail. (2010) 12(12):1300–8. 10.1093/eurjhf/hfq169 20876734

[48] McLellan J Bankhead CR Oke JL Hobbs FDR Taylor CJ Perera R. Natriuretic peptide-guided treatment for heart failure: a systematic review and meta-analysis. BMJ Evid Based Med. (2020) 25(1):33–7. 10.1136/bmjebm-2019-111208 31326896 PMC7029248

[49] Xu T Meng QH Gilchrist SC Lin SH Lin R Xu T Assessment of prognostic value of high-sensitivity cardiac troponin T for early prediction of chemoradiation therapy-induced cardiotoxicity in patients with non-small cell lung cancer: a secondary analysis of a prospective randomized trial. Int J Radiat Oncol Biol Phys. (2021) 111(4):907–16. 10.1016/j.ijrobp.2021.07.035 34302893 PMC8530972

[50] Lyon AR López-Fernández T Couch LS Asteggiano R Aznar MC Bergler-Klein J 2022 ESC guidelines on cardio-oncology developed in collaboration with the European hematology association (EHA), the European society for therapeutic radiology and oncology (ESTRO) and the international cardio-oncology society (IC-OS). Eur Heart J. (2022) 43(41):4229–361. 10.1093/eurheartj/ehac244 36017568

[51] Billebeau G Vodovar N Sadoune M Launay JM Beauvais F Cohen-Solal A. Effects of a cardiac rehabilitation programme on plasma cardiac biomarkers in patients with chronic heart failure. Eur J Prev Cardiol. (2017) 24(11):1127–35. 10.1177/2047487317705488 28452560

[52] Conraads VM Beckers P Vaes J Martin M Van Hoof V De Maeyer C Combined endurance/resistance training reduces NT-proBNP levels in patients with chronic heart failure. Eur Heart J. (2004) 25(20):1797–805. 10.1016/j.ehj.2004.07.022 15474694

[53] Shah ASV Keene SJ Pennells L Kaptoge S Kimenai DM Walker M Cardiac troponins and cardiovascular disease risk prediction: an individual-participant-data meta-analysis. J Am Coll Cardiol. (2025) 85(14):1471–84. 10.1016/j.jacc.2025.02.016 40204376 PMC13266502

[54] Sagris M Antonopoulos AS Angelopoulos A Papanikolaou P Simantiris S Vamvakaris C High-sensitivity troponin (hs-tn) for cardiovascular risk prognostication: a systematic review and meta-analysis. Curr Med Chem. (2024) 31(14):1941–53. 10.2174/0929867330666230315152045 36924099

[55] Venkatesh R Cherlin T, Penn Medicine BioBank, Ritchie MD Guerraty MA Verma SS. Integrating Imaging-Derived Clinical Endotypes with Plasma Proteomics and External Polygenic Risk Scores Enhances Coronary Microvascular Disease Risk Prediction. medRxiv [Preprint]. 2025:2025.08.18.25333844. 10.1101/2025.08.18.25333844 PMC12952676 41758173

[56] Allach Y Barry-Loncq de Jong M Clephas PRD van Gent MWF Brunner-La Rocca HP Szymanski MK Serial cardiac biomarkers, pulmonary artery pressures and traditional parameters of fluid status in relation to prognosis in patients with chronic heart failure: design and rationale of the BioMEMS study. Eur J Heart Fail. (2024) 26(8):1736–44. 10.1002/ejhf.3303 38825743

[57] Mohebi R Liu Y Myhre PL Felker GM Prescott MF Piña IL Heart failure phenotypes according to natriuretic peptide trajectory following initiation of sacubitril/valsartan. JACC Heart Fail. (2023) 11(7):855–8. 10.1016/j.jchf.2023.03.006 37115131

[58] Khan MS Greene SJ DeVore AD. Serial NT-proBNP measurements and implementation of guideline-directed medical therapy. JACC Heart Fail. (2024) 12(3):488–91. 10.1016/j.jchf.2024.01.003 38448150

[59] O'Donoghue ML Morrow DA Cannon CP Jarolim P Desai NR Sherwood MW Multimarker risk stratification in patients with acute myocardial infarction. J Am Heart Assoc. (2016) 5(5):e002586. 10.1161/JAHA.115.002586 27207959 PMC4889163

[60] Gürgöze MT van Vark LC Baart SJ Kardys I Akkerhuis KM Manintveld OC Multimarker analysis of serially measured GDF-15, NT-proBNP, ST2, GAL-3, cTnI, creatinine, and prognosis in acute heart failure. Circ Heart Fail. (2023) 16(1):e009526. 10.1161/CIRCHEARTFAILURE.122.009526 36408685 PMC9833118

[61] DeGroat W Abdelhalim H Patel K Mendhe D Zeeshan S Ahmed Z. Discovering biomarkers associated and predicting cardiovascular disease with high accuracy using a novel nexus of machine learning techniques for precision medicine. Sci Rep. (2024) 14(1):1. 10.1038/s41598-023-50600-8 38167627 PMC10762256

[62] Ambale-Venkatesh B Yang X Wu CO Liu K Hundley WG McClelland R Cardiovascular event prediction by machine learning: the multi-ethnic study of atherosclerosis. Circ Res. (2017) 121(9):1092–101. 10.1161/CIRCRESAHA.117.311312 28794054 PMC5640485

[63] Krittanawong C Zhang H Wang Z Aydar M Kitai T. Artificial intelligence in precision cardiovascular medicine. J Am Coll Cardiol. (2017) 69(21):2657–64. 10.1016/j.jacc.2017.03.571 28545640

[64] Johnson KW Torres Soto J Glicksberg BS Shameer K Miotto R Ali M Artificial intelligence in cardiology. J Am Coll Cardiol. (2018) 71(23):2668–79. 10.1016/j.jacc.2018.03.521 29880128

[65] Carrasco-Ribelles LA Llanes-Jurado J Gallego-Moll C Cabrera-Bean M Monteagudo-Zaragoza M Violán C Prediction models using artificial intelligence and longitudinal data from electronic health records: a systematic methodological review. J Am Med Inform Assoc. (2023) 30(12):2072–82. 10.1093/jamia/ocad168 37659105 PMC10654870

[66] Moglia V Johnson O Cook G de Kamps M Smith L. Artificial intelligence methods applied to longitudinal data from electronic health records for prediction of cancer: a scoping review. BMC Med Res Methodol. (2025) 25(1):24. 10.1186/s12874-025-02473-w 39875808 PMC11773903

[67] Nguena Nguefack HL Pagé MG Katz J Choinière M Vanasse A Dorais M Trajectory modelling techniques useful to epidemiological research: a comparative narrative review of approaches. Clin Epidemiol. (2020) 12:1205–22. 10.2147/CLEP.S265287 33154677 PMC7608582

[68] Drouard G Mykkänen J Heiskanen J Pohjonen J Ruohonen S Pahkala K Exploring machine learning strategies for predicting cardiovascular disease risk factors from multi-omic data. BMC Med Inform Decis Mak. (2024) 24(1):116. 10.1186/s12911-024-02521-3 38698395 PMC11064347

[69] Noseworthy PA Attia ZI Behnken EM Giblon RE Bews KA Liu S Artificial intelligence-guided screening for atrial fibrillation using electrocardiogram during sinus rhythm: a prospective non-randomised interventional trial. Lancet. (2022) 400(10359):1206–12. 10.1016/S0140-6736(22)01637-3 36179758

[70] Weng SF Reps J Kai J Garibaldi JM Qureshi N. Can machine-learning improve cardiovascular risk prediction using routine clinical data? PLoS One. (2017) 12(4):e0174944. 10.1371/journal.pone.0174944 28376093 PMC5380334

[71] Alaa AM Bolton T Di Angelantonio E Rudd JHF van der Schaar M. Cardiovascular disease risk prediction using automated machine learning: a prospective study of 423,604 UK biobank participants. PLoS One. (2019) 14(5):e0213653. 10.1371/journal.pone.0213653 31091238 PMC6519796

[72] Ye C Fu T Hao S Zhang Y Wang O Jin B Prediction of incident hypertension within the next year: prospective study using statewide electronic health records and machine learning. J Med Internet Res. (2018) 20(1):e22. 10.2196/jmir.9268 29382633 PMC5811646

[73] Ward A Sarraju A Chung S Li J Harrington R Heidenreich P Machine learning and atherosclerotic cardiovascular disease risk prediction in a multi-ethnic population. NPJ Digit Med. (2020) 3:125. 10.1038/s41746-020-00331-1 33043149 PMC7511400

[74] Burrello J Gallone G Burrello A Jahier Pagliari D Ploumen EH Iannaccone M Prediction of all-cause mortality following percutaneous coronary intervention in bifurcation lesions using machine learning algorithms. J Pers Med. (2022) 12(6):990. 10.3390/jpm12060990 35743777 PMC9224705

[75] Shmatko A Jung AW Gaurav K Brunak S Mortensen LH Birney E Learning the natural history of human disease with generative transformers. Nature. (2025) 647(8088):248–56. 10.1038/s41586-025-09529-3 40963019 PMC12589094

[76] Climente-González H Oh M Chajewska U Hosseini R Mukherjee S Gan W Interpretable machine learning leverages proteomics to improve cardiovascular disease risk prediction and biomarker identification. Commun Med (Lond). (2025) 5(1):170. 10.1038/s43856-025-00872-0 40389651 PMC12089484

[77] Kim MS Khurshid S Kany S Weng LC Urbut S Roselli C Machine learning-based plasma protein risk score improves atrial fibrillation prediction over clinical and genomic models. Circ Genom Precis Med. (2025) 18(4):e004943. 10.1161/CIRCGEN.124.004943 40525300 PMC12257488

[78] Nielsen SD Dobrosavljevic M Andell P Chang Z Clemmensen LKH Larsson H Development and external validation of machine learning approaches for risk prediction of cardiovascular disease in individuals with schizophrenia: a nationwide Swedish and Danish study. BMJ Ment Health. (2026) 29(1):e301964. 10.1136/bmjment-2025-301964 41545227 PMC12815116

[79] Collins GS Moons KGM Dhiman P Riley RD Beam AL Van Calster B TRIPOD + AI statement: updated guidance for reporting clinical prediction models that use regression or machine learning methods. Br Med J. (2024) 385:e078378. 10.1136/bmj-2023-078378 38626948 PMC11019967

[80] Moons KGM Damen JAA Kaul T Hooft L Andaur Navarro C Dhiman P PROBAST + AI: an updated quality, risk of bias, and applicability assessment tool for prediction models using regression or artificial intelligence methods. Br Med J. (2025) 388:e082505. 10.1136/bmj-2024-082505 40127903 PMC11931409

[81] Chen RJ Wang JJ Williamson DFK Chen TY Lipkova J Lu MY Algorithmic fairness in artificial intelligence for medicine and healthcare. Nat Biomed Eng. (2023) 7(6):719–42. 10.1038/s41551-023-01056-8 37380750 PMC10632090

[82] Sahiner B Chen W Samala RK Petrick N. Data drift in medical machine learning: implications and potential remedies. Br J Radiol. (2023) 96(1150):20220878. 10.1259/bjr.20220878 36971405 PMC10546450

[83] Kappen TH van Klei WA van Wolfswinkel L Kalkman CJ Vergouwe Y Moons KGM. Evaluating the impact of prediction models: lessons learned, challenges, and recommendations. Diagn Progn Res. (2018) 2:11. 10.1186/s41512-018-0033-6 31093561 PMC6460651

[84] Apple FS. Counterpoint: standardization of cardiac troponin I assays will not occur in my lifetime. Clin Chem. (2012) 58(1):169–71. 10.1373/clinchem.2011.166165 21940657

[85] Cemin R Daves M. Pre-analytic variability in cardiovascular biomarker testing. J Thorac Dis. (2015) 7(10):E395–401. 10.3978/j.issn.2072-1439.2015.10.03 26623116 PMC4635305

[86] Hamm CW Giannitsis E Katus HA. Cardiac troponin elevations in patients without acute coronary syndrome. Circulation. (2002) 106(23):2871–2. 10.1161/01.cir.0000044342.50593.63 12460862

[87] Goodacre S Thokala P. The economics of cardiac biomarker testing in suspected myocardial infarction. Clin Biochem. (2015) 48(4-5):213–7. 10.1016/j.clinbiochem.2014.08.012 25173750

[88] de Gonzalo-Calvo D Pérez-Boza J Curado J Devaux Y, EU-CardioRNA COST Action CA17129. Challenges of microRNA-based biomarkers in clinical application for cardiovascular diseases. Clin Transl Med. (2022) 12(2):e585. 10.1002/ctm2.585 35167732 PMC8846372

[89] Zhang B Schmidlin T. Recent advances in cardiovascular disease research driven by metabolomics technologies in the context of systems biology. NPJ Metab Health Dis. (2024) 2(1):25. 10.1038/s44324-024-00028-z 40603608 PMC12118667

[90] Januzzi JL Jr Chen-Tournoux AA Christenson RH Doros G Hollander JE Levy PD N-Terminal pro-B-type natriuretic peptide in the emergency department: the ICON-RELOADED study. J Am Coll Cardiol. (2018) 71(11):1191–200. 10.1016/j.jacc.2018.01.021 29544601

[91] Vasudevan S Saha A Tarver ME Patel B. Digital biomarkers: convergence of digital health technologies and biomarkers. NPJ Digit Med. (2022) 5(1):36. 10.1038/s41746-022-00583-z 35338234 PMC8956713

[92] Wells QS Gupta DK Smith JG Collins SP Storrow AB Ferguson J Accelerating biomarker discovery through electronic health records, automated biobanking, and proteomics. J Am Coll Cardiol. (2019) 73(17):2195–205. 10.1016/j.jacc.2019.01.074 31047008 PMC6501811

[93] Felker GM Anstrom KJ Adams KF Ezekowitz JA Fiuzat M Houston-Miller N Effect of natriuretic peptide-guided therapy on hospitalization or cardiovascular mortality in high-risk patients with heart failure and reduced ejection fraction: a randomized clinical trial. JAMA. (2017) 318(8):713–20. 10.1001/jama.2017.10565 28829876 PMC5605776

