# Supplementary material for: From acute diagnosis to longitudinal risk stratification: a paradigm shift in the clinical role of cardiac biomarkers
Source: Front Cardiovasc Med. 2026 Mar 13;13:1778456. doi: 10.3389/fcvm.2026.1778456 (PMC13021771; doi:10.3389/fcvm.2026.1778456)
Supplement: Supplementary file 1 [file Table1.docx]

**Supplementary Table 1. Representative Cardiac Biomarkers Across Disease Stages and Clinical Applications**

| Disease stage | Biomarker category | Representative biomarkers | Pathophysiological relevance | Primary clinical application |
| --- | --- | --- | --- | --- |
| High-risk screening & primary prevention | Myocardial injury/inflammation | hs-cTn, hs-CRP | Subclinical cardiomyocyte injury and low-grade systemic inflammation | Identification of high-risk individuals; long-term cardiovascular risk prediction |
| Subclinical disease detection | Fibrosis/remodeling /stress response | sST2, Galectin-3, PIIINP, GDF-15 | Myocardial fibrosis, extracellular matrix remodeling, early stress signaling | Early detection of structural and functional cardiac abnormalities; identification of preclinical heart failure |
| Acute cardiovascular events | Myocardial injury markers | hs-cTn, cMyC | Acute cardiomyocyte necrosis and early myocardial injury | Rapid diagnosis and differential diagnosis of acute myocardial infarction; emergency triage |
| In-hospital and post-discharge risk stratification | Injury/hemodynamic stress/inflammation (multi-marker) | hs-cTn, BNP/NT-proBNP, hs-CRP | Integrated assessment of myocardial injury, wall stress, and inflammatory burden | Prognostic stratification; prediction of mortality and rehospitalization risk |
| Chronic disease management | Hemodynamic stress / injury/ fibrosis | BNP/NT-proBNP, hs-cTn, sST2 | Persistent myocardial stress, ongoing injury, and adverse remodeling | Therapy guidance and monitoring; assessment of treatment response and disease progression |
| Cardiotoxicity monitoring & rehabilitation | Injury /hemodynamic stress | hs-cTn, BNP/NT-proBNP | Early myocardial injury and stress related to chemotherapy or exercise adaptation | Early detection of cardiotoxicity; optimization of cardioprotective strategies and rehabilitation programs |

**Notes**: hs-cTn, high-sensitivity cardiac troponin; hs-CRP, high-sensitivity C-reactive protein; sST2, soluble suppression of tumorigenicity 2; PIIINP, procollagen type III N-terminal propeptide; GDF-15, growth differentiation factor 15; cMyC, cardiac myosin-binding protein C; BNP, B-type natriuretic peptide; NT-proBNP, N-terminal pro–B-type natriuretic peptide.
